# Intracranial Sonodynamic Therapy With 5-Aminolevulinic Acid and Sodium Fluorescein: Safety Study in a Porcine Model

**DOI:** 10.3389/fonc.2021.679989

**Published:** 2021-06-21

**Authors:** Luca Raspagliesi, Antonio D’Ammando, Matteo Gionso, Natasha D. Sheybani, Maria-Beatriz Lopes, David Moore, Steven Allen, Jeremy Gatesman, Edoardo Porto, Kelsie Timbie, Andrea Franzini, Francesco Di Meco, Jason Sheehan, Zhiyuan Xu, Francesco Prada

**Affiliations:** ^1^ Neurosurgery Department, Fondazione IRCCS Istituto Neurologico Carlo Besta, Milan, Italy; ^2^ Humanitas University, Pieve Emanuele, Italy; ^3^ Division of Oncology, Department of Medicine, Stanford Cancer Institute, Stanford University, Stanford, CA, United States; ^4^ Department of Pathology, University of Virginia, Charlottesville, VA, United States; ^5^ Focused Ultrasound Foundation, Charlottesville, VA, United States; ^6^ Department of Biomedical Engineering, University of Virginia, Charlottesville, VA, United States; ^7^ Center for Comparative Medicine, University of Virginia, Charlottesville, VA, United States; ^8^ Department of Health Sciences, University of Milan, Milan, Italy; ^9^ Department of Neurosurgery, Humanitas Clinical and Research Center, Milan, Italy; ^10^ Department of Neurological Surgery, Johns Hopkins Medical School, Baltimore, MD, United States; ^11^ Department of Neurological Surgery, University of Virginia, Charlottesville, VA, United States; ^12^ Acoustic Neuroimaging and Therapy Laboratory, Fondazione IRCCS Istituto Neurologico Carlo Besta, Milan, Italy

**Keywords:** safety, brain tumors, ultrasound, sonodynamic therapy (SDT), fluorescein (FL), fluorescein 5-aminolevulinic acid (5-ALA), focused ultrasound (FUS)

## Abstract

**Background:**

Sonodynamic therapy (SDT) is an emerging ultrasound-based treatment modality for malignant gliomas which combines ultrasound with sonosensitizers to produce a localized cytotoxic and modulatory effect. Tumor-specificity of the treatment is achieved by the selective extravasation and accumulation of sonosensitizers in the tumor-bearing regions. The aim of this study is to demonstrate the safety of low-intensity ultrasonic irradiation of healthy brain tissue after the administration of FDA-approved sonosensitizers used for SDT in experimental studies in an *in vivo* large animal model.

**Methods:**

In vivo safety of fluorescein (Na-Fl)- and 5 aminolevulinic acid (5-ALA)-mediated low-intensity ultrasound irradiation of healthy brain parenchyma was assessed in two sets of four healthy swine brains, using the magnetic resonance imaging (MRI)-guided Insightec ExAblate 4000 220 kHz system. After administration of the sonosensitizers, a wide fronto-parietal craniotomy was performed in pig skulls to allow transmission of ultrasonic beams. Sonication was performed on different spots within the thalamus and periventricular white matter with continuous thermal monitoring. Sonication-related effects were investigated with MRI and histological analysis.

**Results:**

Post-treatment MRI images acquired within one hour following the last sonication, on day one, and day seven did not visualize any sign of brain damage. On histopathology, no signs of necrosis or apoptosis attributable to the ultrasonic treatments were shown in target areas.

**Conclusions:**

The results of the present study suggest that either Na-FL or 5-ALA-mediated sonodynamic therapies under MRI-guidance with the current acoustic parameters are safe towards healthy brain tissue in a large *in vivo* model. These results further support growing interest in clinical translation of sonodynamic therapy for intracranial gliomas and other brain tumors.

## Introduction

High-grade gliomas (HGGs) are the most common and aggressive group of primary tumors of the brain deriving from glial cells, with an incidence of 3-5 cases per 100,000 inhabitants in the United States (1, 2). These tumors are characterized by an infiltrative and diffuse nature, which results in unavoidable early recurrences and a poor overall survival (2, 3). Indeed, current therapeutic schemes, often involving maximal surgical resection, subsequent irradiation and cytotoxic chemotherapy, have little influence on the outcome of HGGs, due to chemo- and radio-resistance of tumor stem cells, rapid infiltration of tumor cells into normal brain tissue through axonal pathways, and low chemotherapy penetration through intact blood-brain barrier (BBB) in the peritumoral region, where tumor stem cells often reside (4–6).

Over the years, the unsatisfactory yield of existing treatments has prompted the search for new therapeutic approaches to HGGs (7). Among investigated techniques is sonodynamic therapy (SDT), which has proven in recent years to be a promising approach for treatment of intracranial tumors. It relies on the natural pharmacokinetics and tumor-selectivity of non-toxic sound-sensitive molecules, called sonosensitizers, which are able to locally magnify the cytotoxic and modulatory effects of low-frequency low-intensity ultrasound waves (8). Possible mechanisms of SDT include peroxidation of membrane lipids *via* peroxyl radicals, generated by activation of the sonosensitizer; physical destabilization of the cell membrane, allowing increased susceptibility of the cell to shear forces; and enhanced uptake of chemotherapy due to sonoporation (9). There are many different agents known to be effective sonosensitizers for SDT; the most common are porphyrin-based or xanthene-based. The ideal sonosensitizer should have no substantial *in vivo* toxicity, high selectivity for the target lesion, and a high clearance rate from healthy tissue (10).

The concept that porphyrin-based molecules could be used as sonosensitizers dates back to the employment of hematoporphyrin in photodynamic therapy and is based on the evidence that electronic excitations of this compound by ultrasound energy initiate a chemical process that eventually results in the formation of cytotoxic reactive oxygen species (ROS) (11). 5-aminolevulinic acid (5-ALA) is a porphyrin-based compound, often employed in SDT for glioma model due to its characteristic selectivity for tumor cells. In particular, 5-ALA is implicated in physiological heme synthesis and normally does not produce ROS. However, when an exogenous source of 5-ALA is administered, one of its deriving porphyrins - Protoporphyrin IX (PpIX) - accumulates in the intracellular compartment of tumor cells and, when activated by low-intensity ultrasound, generates cytotoxic ROS that in turn damage target cells (12). The selective accumulation of PpIX in HGG, but not in normal tissue, is explained by a variety of different mechanisms: the limited activity of ferrochelatase, an enzyme for PpIX metabolism to heme, in tumor cells; the increased capacity for converting 5-ALA to PpIX; enhanced uptake of the compound resulting from impairment of the BBB surrounding glial tumors (13–15). Due to this highly selective accumulation in glioma cells, 5-ALA is, to date, the most commonly investigated sonosensitizer for glioma-SDT (4).

Sodium Fluorescein (Na-Fl) is an organic xanthene-based compound often used as a fluorescent dye during surgical removal of malignant gliomas. Its selectivity for pathologic tissue after systemic administration is achieved through preferential accumulation in brain areas with impaired BBB and rapid washout from vessels and healthy tissue (16, 17). After intravenous administration, Na-Fl weakly binds to blood proteins; hence, the presence of both bound (66 kDa) and unbound protein (376 Da) in circulation. The latter is able to cross normal BBB and readily penetrate normal brain tissue, wherein its concentration is highly time-dependent and peaks between 15 and 30 minutes after administration (17).

Intrinsic characteristics of Na-Fl (i.e. high extinction coefficient and high fluorescence quantum yield in water) account for its photodynamic and sonodynamic activity when appropriately stimulated. In particular, ultrasonic irradiation of fluorescein results in the generation of singlet oxygen (^1^O_2_), which induces oxidative stress and resultantly injures surrounding tissue (18). Indeed, Na-Fl has only recently been investigated as a sonosensitizer for glioma-SDT, exhibiting efficacy and safety in an ectopic model of rat glioma (19).

Many preclinical studies have already investigated the efficacy of both 5-ALA and Na-Fl in treating gliomas. However, studies published to date have only employed small animal (i.e. rodent) models, typically with minor subsets of healthy subjects, and have been focused on tumor response, lending minimal insight into the effects of SDT on the surrounding brain parenchyma (4). Furthermore, sonication devices were mostly experimental, with only one study having employed a device currently in use for ultrasound therapy in humans (20). The objective of the present study is to demonstrate the feasibility and safety of SDT in a large animal model using both 5-ALA and Na-Fl as sonosensitizers for *in vivo* application of SDT to healthy pigs using an MRI-guided FUS (MRgFUS) device (Insightec 220 kHz MRgFUS system).

## Materials and Methods

### Study Design

**Figure 1:**
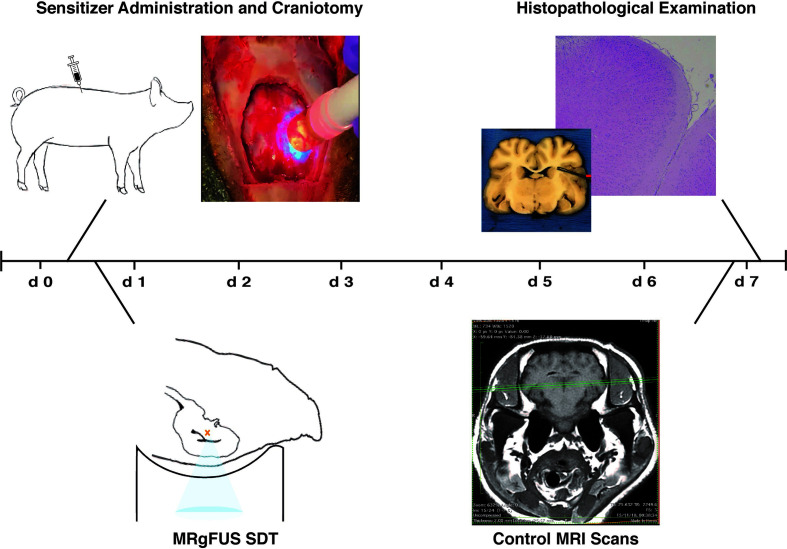
Timeline of experimental treatments. Subjects were administered the assigned sensitizing compound right before undergoing craniotomy at day one. After the procedure, subjects were placed in prone position inside the Exablate device and received MRI-guided low-intensity insonation. On the day 3, MRI scans were acquired again to be later compared with pre-treatment tracking images. Upon sacrifice on the seventh day, brains were harvested for histopathological examination.

Healthy adult pigs (Sus scrofa domesticus) underwent MRI-guided cerebral sonodynamic therapy. One animal was preliminarily employed as a test-pilot to assess workflow and feasibility and was sacrificed the same day of the procedure. Experimental subjects were randomized into two treatment arms - SDT with 5-ALA (n=3) and SDT with Na-Fl (n=5) - as summarized in **Table 1**. Subjects received the assigned sonosensitizer and underwent a craniotomy procedure and subsequent sonication *via* the MRgFUS system (ExAblate, Tirat Carmel, Israel). Ultrasound waves were focused on two target structures within a single cerebral hemisphere, employing the contralateral hemisphere of each subject as a direct internal control. Subjects underwent MRI imaging at day 1 and at day 7 after the procedure; at day 7 subjects received a second dose of the assigned sonosensitizer and, two to three hours later, they were euthanized; after sacrifice, subjects’ brains were extracted and fixed for histopathological assessment. **Figure 1** summarizes the timeline of experimental treatments.

**Table 1:** The table summarizes the subjects employed in the present studies, exemplifying the sonosensitizer administered, the areas of sonication, the number of spots sonicated, and the post-procedure scans performed.

#	Group	Mod	Areas	Locations	Sonications	Sub-spots	Post-procedure scans
**1**	Pilot	na	fPWM	1	6	4	na
			Th	/	/	/	
**2**	5-ALA	94	fPWM	2	6	4	Cube T2; T1
			Th	2	6	4	
**3**	5-ALA	95	fPWM	2	6	10	Sag Cube FLAIR Sag CUBE T2 Sag CUBE T1
			Th	2	6	10	
**4**	5-ALA	95	fPWM	2	6	10	Sag Cube FLAIR Sag CUBE T2 Sag CUBE T1
			Th	2	6	10	
**5**	Na-FL	95	fPWM	2	6	10	Sag Cube FLAIR Sag CUBE T2 Sag CUBE T1
			Th	2	6	10	
**6**	Na-FL	OMISSIS (95)	fPWM	na	na	na	na
			Th	na	na	na	
**7**	Na-FL	95	fPWM	2	6	10	Sag Cube FLAIR Sag CUBE T2 Sag CUBE T1
			Th	2	6	10	
**8**	Na-FL	12	fPWM	2	7	10	Sag Cube FLAIR Sag CUBE T2 Sag CUBE T1
			Th	2	7	10	
**9**	Na-FL	12	fPWM	2	7	10	Sag Cube FLAIR Sag CUBE T2 Sag CUBE T1
			Th	2	7	10	

*fPWM*, frontal Periventricular White Matter; *Th*, Thalamus; *na*, not available.

### Subjects’ Preparation

All procedures were performed at University of Virginia strictly following Animal Care and Use Committee (ACUC) guidelines (protocol approval #4152).

Two to three hours prior to the acoustic sonications, each subject received a full dose of either 5-ALA (20mg/Kg body weight) or Na-Fl (10mg/Kg body weight), namely the dose currently used in clinical practice for fluorescence-guided surgery, through the left lateral auricular vein with a peripheral venous line.

### Surgical Procedure

Due to the peculiar anatomy of the porcine skull, which is not compatible with the geometry of the ExAblate’s stereotaxic helmet, a craniotomy procedure was necessary to allow optimal coupling of brain tissues with the device. After administration of the sonosensitizer, subjects were brought to the veterinary facility at University of Virginia and positioned on the surgical table in a prone position.

Each subject subsequently received general anesthesia with Telazol/Xylazine 4-6/2 mg per kg intramuscularly (induction) and Isoflurane (maintenance).

After induction of anesthesia, the skin was epilated and disinfected with betadine and alcohol 70% and the sterile surgical field was prepared. The procedure began with a 35mm incision of the skin 10 mm from the midline and 10 mm anterior to the coronal suture and dissection of the subcutaneous tissues. Once the bone was exposed, a 5 cm-diameter wide fronto-parietal craniotomy was performed with rongeurs and a cutting burr to expose the region of brain superficial to the selected targets. Subsequently, sterile water was inserted into the cavity and the skin was closed over the brain without the interposition of the bone flap to allow the unhindered penetration of the ultrasound waves.

### Sonication Procedure

Each subject was subsequently transported to the ExAblate 220 kHz MRgFUS device located at the University of Virginia Focused Ultrasound Center, secured to a helmet and positioned supine on the MRI table of the device while maintained under general anesthesia.

**Figure 2:**
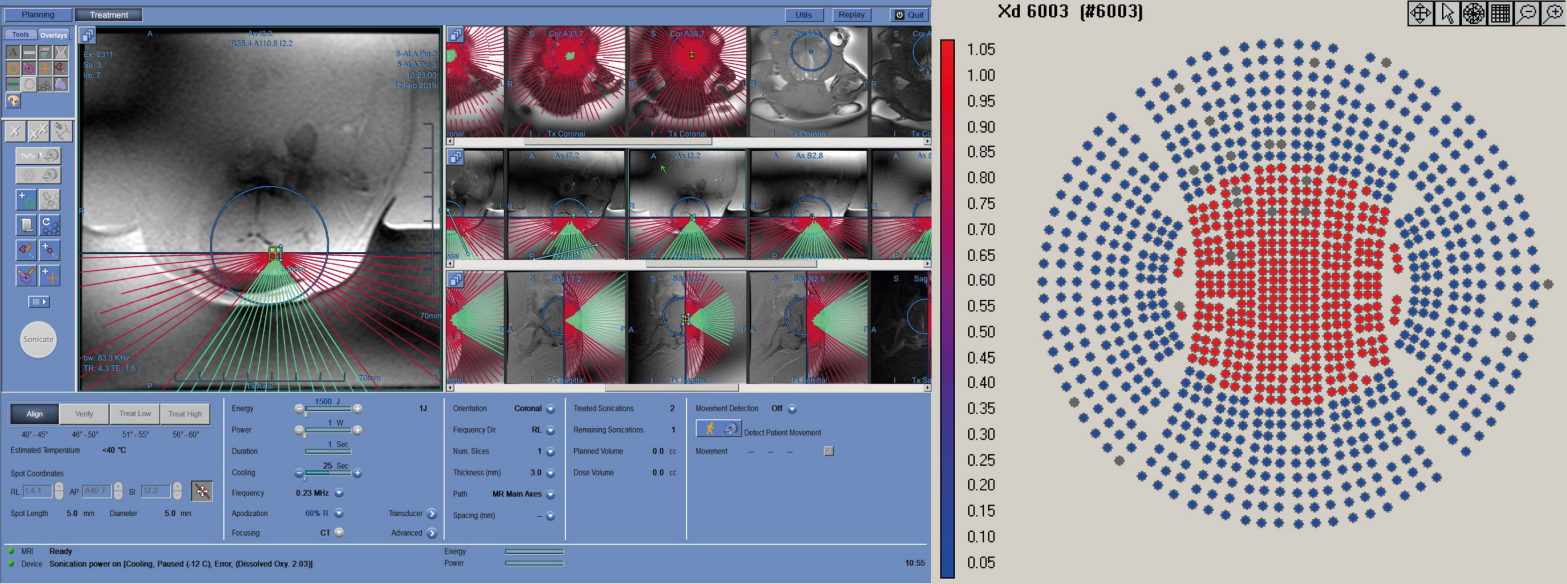
T1- and T2-weighted images acquired after the sonication procedure.

Treatment targets were identified on the MRI using a 2D, multi-slice, balanced, steady-state acquisition (FIESTA) with whole-brain coverage. The sequence was repeated with prescribed slices oriented in the coronal, sagittal, and transverse orientation. Slices were prescribed to cover the entire brain volume. The resulting images presented strong contrast between gray/white matter and the ventricles, allowing stereotactic planning of the treatment targets. Conditions that might compromise the results, e.g. pre-existing lesions, inflammation or edema, were then ruled out (**Figure 2**).

**Figure 3:**
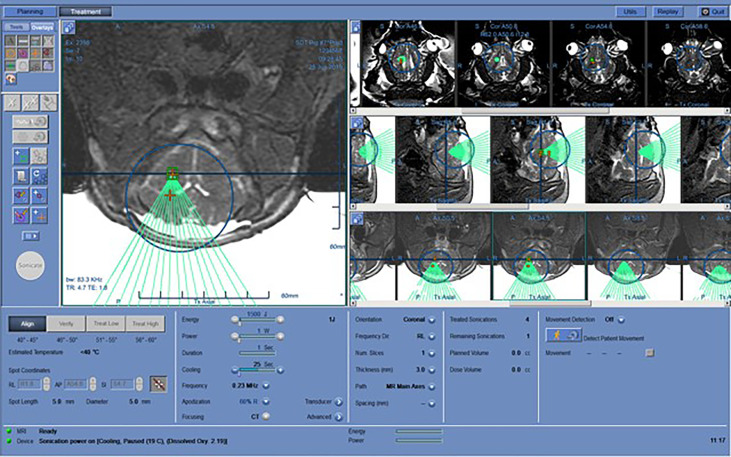
Working layout of the Exablate system during insonation of one subject. The insonation pattern is superimposed on the pre-acquired MR images in different slices.

The FUS treatment was administered with a power level of 1 W (0.57 acoustic power) to achieve a focused peak power of 2-3 W/cm^2^. Peak pressure for this power level, measured with a hydrophone, was around 200 kPa. In order to maximize the treatment volume, we leveraged the 10% duty cycle (DC) of the ExAblate system chosen for the treatment sonication; the device’s multi-element array has the ability to rapidly switch the sonication target by changing the phase of the 1024 elements in the transducer to move the focus of the transducer within a region roughly 2 cm in diameter (**Figure 3**). A sonication pattern was designed to raster the focus across 10 locations centered around the target location on the clinical software. As a result, each insonated region encompassed a rectangle roughly 10x10x15 mm, about the same size and shape of two dice stacked on top of one another with the center of the sonication target located between the two faces of the dice.

### Post-Treatment Procedures and Examination

Immediately after the insonation, each subject was removed from the Insightec system and placed prone in an 8-channel imaging coil. MRI images (T1 and T2-weighted SPACE, 3D-MPRAGE, Diffusion-weighted MRI) were then acquired; diffusion-weighted sequences were considered particularly significant as an early indicator of post-treatment lesion (21). Contrast-enhanced T1 scans were not considered due to the lack of a pathological lesion to produce extravasation of Gadolinium, nor the necessity to asses contrast extravasation as for blood brain barrier opening. Acquisition parameters are described in **Table 2**.

**Table 2:** MRI acquisition parameters used immediately after insonation and at 7-day follow-up imaging.

Scan Type	TR (s)/TE (ms)/TI (ms)	FOV (mm)/Resolution (mm)	Bandwidth (kHz)/Echo train length	b-value s mm^-1^/Directions
**3D T1-w SPACE**	0.6/15.8	160 x 160 x 272/0.5 x 0.5 x 1	244/28	
**3D T2-w SPACE**	3/109	160 x 160 x 272/0.5 x 0.5 x 1	244/130	
**3D-MPRAGE**	1.2/4.1/450	160 x 160 x 272/0.5 x 0.5 x 1	122/32	
**DW**	8/69	160 x 160 x 64/1.25 x 1.25 x 4	1953/128	1000/25

Preliminarily, the test-pilot subject (pig #1), which had received 5-ALA administration, was euthanized immediately after completion of the FUS procedure in order to verify the feasibility of the present experimental protocol.

Conversely, all the other subjects were transported back to the animal nursery unit at University of Virginia after the procedure to be awakened and closely monitored. These subjects underwent further MRI scans at day 7 and were subsequently euthanized. Two to three hours prior euthanasia, each subject received a second dose of the assigned sensitizer (either 5-ALA, 20mg/Kg body weight, or Na-Fl, 10mg/Kg body weight), through the left lateral auricular vein with a peripheral venous line; the purpose of this procedure was to reproduce, at the ex vivo examination, the same parenchymal concentration of the compounds that was present at the time of the sonication.

Upon euthanasia (Euthasol 1ml/10lbs body weight, IV), the brains of all subjects were harvested for histopathological examination.

### Histopathological Examination

After being harvested, brains were preserved in 10% neutral buffered formalin (Sigma-Aldrich, USA) for histological analysis.

Brains were then cut in coronal sections; anatomical slices containing treated areas and surrounding regions were paired with corresponding intra-procedural MRI slices for comparison purposes.

Slices corresponding to target locations were first macroscopically inspected to identify any gross signs of damage resulting from sonication procedures. Subsequently, tissue sections from the bilateral thalami and periventricular areas were then sampled and processed for paraffin-embedding. Microscopic examination was performed on 5 μm-thick histologic sections stained by hematoxylin and eosin.

### Fluorescence Quantification

The tip of the frontal lobe from the pigs’ extracted brains were excised and employed to quantify PpIX and NA-Fl concentration, respectively.

- *5-ALA group*: the tip was minced into tiny pieces with sterile scissors and weighed. The brain tissue was suspended in 300ul Solvable (Sigma-Aldrich, USA) in a 45°C water bath for 15 minutes. Meanwhile, a final concentration of 200uM PpIX powder (purchased from Sigma-Aldrich, USA) in Solvable was prepared, and then serial dilutions of PpIX were prepared in 200ul Solvable in a 96-well assay plate (Costar, black plate, clear bottom with lid, Corning, USA) for a standard curve of PpIX. Transferred 200ul supernatant from the aforementioned brain tissue preparation tube. The fluorescence intensity was quantitated using SpectraMax iD3 multi-mode (Fluorescence, luminescence, absorbance) plate reader with the excitation wavelength of 405 nm and the emission wavelength of 635 nm (Molecular Devices, Biomolecular Analysis Facility, University of Virginia) (22).
- *Na-Fl group*: excised regions of cerebral tissue were immersed in a phosphate buffered saline bath and imaged using the IVIS Spectrum (PerkinElmer) for Na-FL fluorescence signal using 494/521 excitation/emission peak inputs and auto-exposure settings. The Living Images software package (PerkinElmer) was used for quantification of epifluorescence from images. Identical circular Regions of Interest (ROI) were applied to encompass individual tissues or a background region, following which Na-Fl fluorescence was quantified and reported as radiance.

## Results

Out of 9 total experiments performed, 1 subject (pig #6) was excluded from analysis since, during the closure of the scalp over the craniotomy, it suffered a direct injury to the non-sonicated hemisphere; histopathological examination showed a superficial subacute infarct with organizing subarachnoid and intraparenchymal hemorrhage, surrounding infarction of the cortex and significant inflammatory reaction comprised of lymphocytes, macrophages and eosinophils, while the left cortex showed focal organizing subarachnoid hemorrhage. Although ultimately excluded, this subject went through the whole experimental protocol until sacrifice on the seventh day. In the remaining subjects, the protocol was carried out without any relevant complications. Of note, during the sonication procedure, pig #8 was reported to have moved from reference position before completion of the treatment due to suboptimal fixation of the helmet. Nevertheless, after being repositioned, the treatment scheme was reinitiated, but the subject was ultimately not included in the analysis.

Overall, sonication procedure had a mean duration of 1 hour, 40 minutes, corresponding to 20 minutes for each location.

### Fluorescence Quantification

- *5-ALA group*: fluorescence quantification confirmed the presence of PpIX in all the analyzed specimens. Fluorescence intensity of PpIX was 56,4 μmol/gm brain tissue in pig #2, 7438,4 μmol/gm brain tissue in pig #3 and 8375,5 μmol/gm brain tissue in pig #4. The difference among these results reflected the specific time lapse between the sonosensitizer administration and euthanasia in each subject; the low level of PpIX in subject #1, in particular, might be due to the fact that, due to technical constraints, this specific specimen had to be frozen at -80°C and, then, analyzed 48h after the harvest.
- *Na-Fl group*: epifluorescence imaging revealed noteworthy differential uptake of Na-Fl in cerebral tissues immediately following intervention. The brain exhibited the highest uptake of Na-Fl, approximately 2.1- and 1.2-fold higher than the brainstem and dura, respectively.

### Sonication Procedures and Radiological Findings

In preclinical sonodynamic treatment studies performed to date, typically a 10% DC (where the transducer is on 10% of the time and off for 90%) has been used in order to provide a power sufficient to activate the sonosensitizer agent but avoid a thermal rise in the target and surrounding tissues (23–25). In our procedure, each sonication target was a series of 10 sub-sonications centered around the target chosen on the targeting software; by taking advantage of the fact that each sonication needed to be 10ms in length and a 10% DC, the 90% off time could be used to treat additional targets. So, the total ON-time for the transducer was almost 100%, but each spot received only a 10% DC, as usual. The steering used for each sonication are exemplified in **Table 3**.

**Table 3:** The table shows the steering used for all sonication; coordinates are listed in mm.

Sonication 1	(0.0, 0.0, 3.0)
Sonication 2	(3.0, 3.0, 3.0)
Sonication 3	(-3.0, 3.0, 3.0)
Sonication 4	(-3.0, -3.0, 3.0)
Sonication 5	(3.0, -3.0, 3.0)
Sonication 6	(0.0, 0.0, -3.0)
Sonication 7	(3.0, 3.0, -3.0)
Sonication 8	(-3.0, 3.0, -3.0)
Sonication 9	(-3.0, -3.0, -3.0)
Sonication 10	(3.0, -3.0, -3.0)

**Figure 4:**
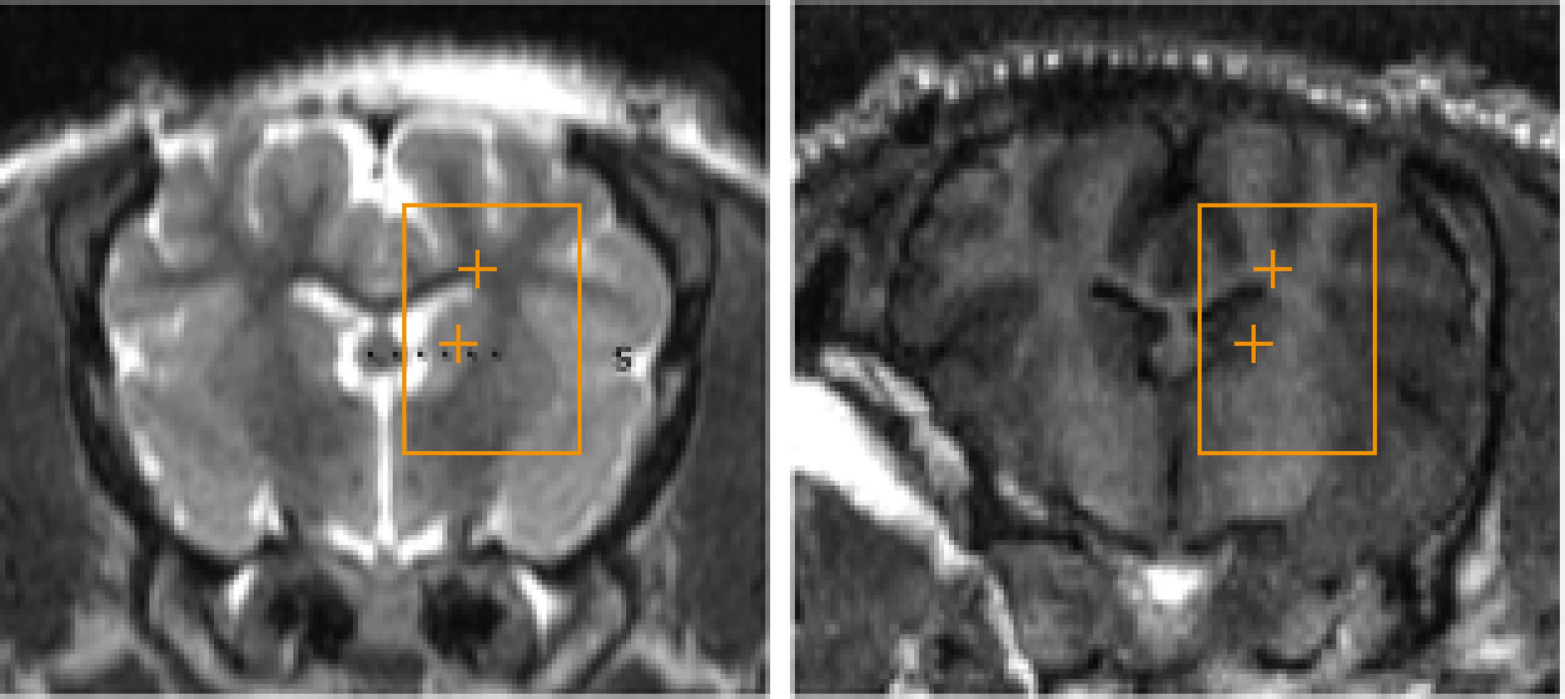
Three-axial representation of the treatment-planning phase prior to insonation. Insonation targets are represented by orange cross signs within Basal Ganglia and Periventricular White Matter. Each location was composed of 10 sub-sonication spots that were alternately targeted by the tracking system to exploit the “off-duty” time of each single insonation.

The spot size for the 220kHz ExAblate system was approximately 4x10mm (full width, half maximum). This yielded a treatment volume roughly 10x10x15 mm in 20’ (**Figure 4**).

In order to sonicate for the full 20 minutes required for the treatment, multiple sonications were required due to a limitation with the treatment software that only allowed a maximum 180 seconds per sonication. Sonications were restarted when each was finished (about 5-10 seconds between each sonication) for 6 times with a final 7^th^ sonication lasting for 120 seconds to give a full 20 minutes. Sonications were typically apodized to about 80% to allow transmission through the craniotomy.

**Figure 5:**
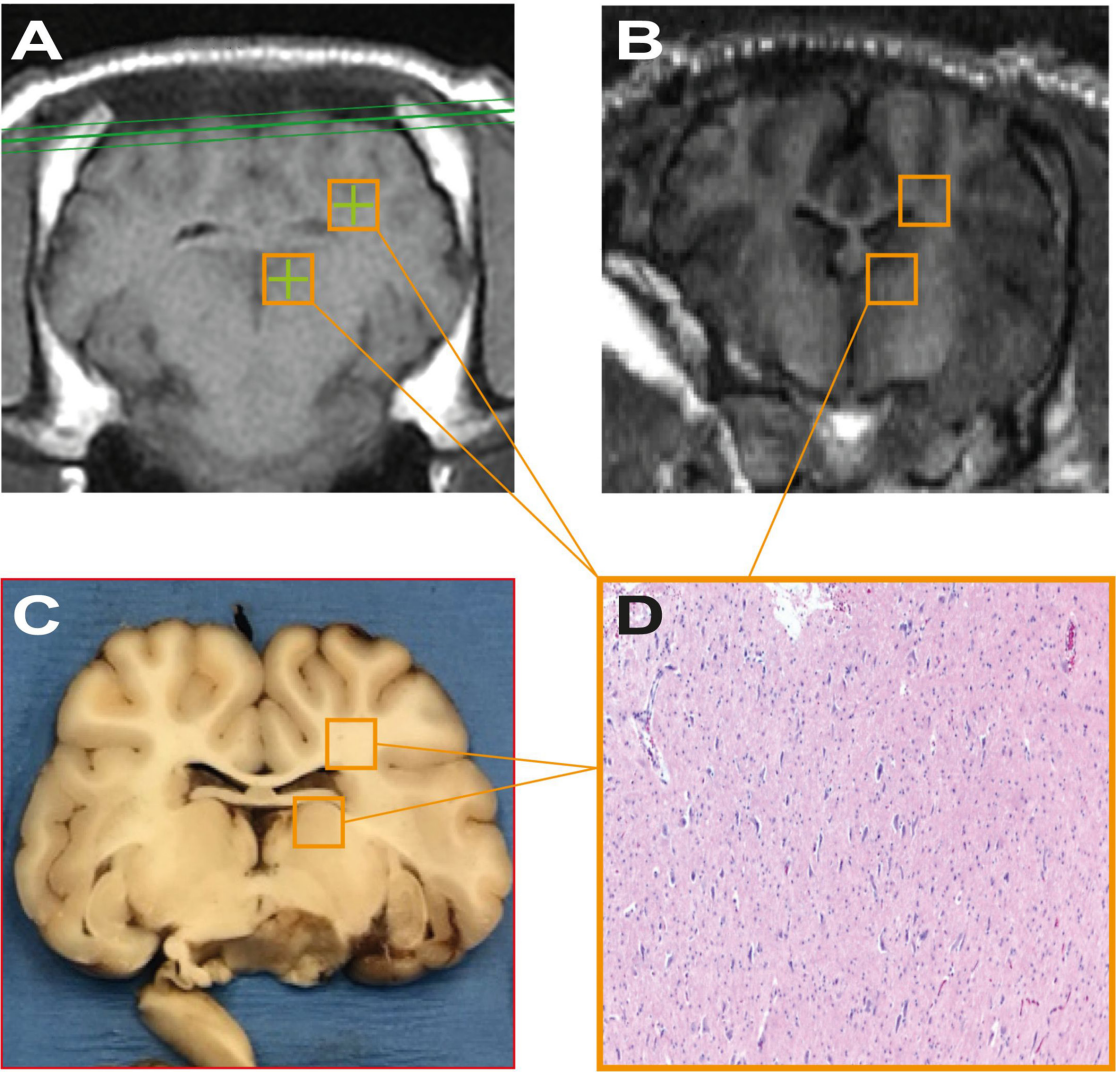
**(A, B)** represent pre-treatment tracking images and post-procedure scans respectively, showing no significant differences attributable to the sonodynamic therapeutic protocol. Similarly, **(C, D)** were acquired during macroscopical examination and histopathological analysis of specimens and show no signs of necrosis or apoptotic phenomena after the treatment.

The radiological appearance of the target regions appeared indistinguishable from normative, healthy tissue on all scans (**Figure 5**). There were no indications of edema or hematoma throughout the whole brain, with the exception of subject #6, who, as mentioned above, suffered an injury during scalp closure.

### Histopathological Findings

- *5-ALA group*: At macroscopic examination, coronal sections did not reveal any gross abnormalities at target areas (bilateral basal ganglia, thalamus, periventricular white matter). Multifocal areas of acute subarachnoid hemorrhage were present in pig #2 and pig #3, suggestive of terminal event. At histopathology, target areas (left thalamus and periventricular white matter) did not show any significant pathologic abnormality, including infarction, necrosis or hemorrhage; neurons and glial cells were intact, with no significant cellular alterations. Of note, microscopic intraparenchymal hemorrhages, without tissue reaction, were found in the lower portions of the thalamus of pig #2 and pig #3, suggestive of terminal event. Contralateral areas, serving as direct controls, were unremarkable both at macro and microscopic examination.
- *Na-FL group*: At macroscopic examination, subjects were completely unremarkable. At histopathology, target areas (left thalamus and periventricular white matter) did not reveal significant pathologic abnormalities including infarction, necrosis or hemorrhage. There was integrity of the neurons and glial cells with no significant cellular alterations. The contralateral areas were also unremarkable. Control areas of pigs #5, #8 and #9 were unremarkable. On the other hand, pig #7 showed organizing subarachnoid hemorrhage with gliosis of the subpial area of the right cortex.

## Discussion

The results of the present study demonstrate the safety of sonicating the healthy brain parenchyma after the administration of sonosensitizing agents in a large *in vivo* model. Moreover, considering the heterogeneous and complex cytoarchitecture of the encephalon, the safety of the procedure was assessed for both grey and white matter structures, represented by the thalamus and frontal periventricular white matter, respectively, demonstrating that neither of these areas are subjected to macro- and microscopic damage when treated with SDT. The presence of the sensitizing compounds within insonated brains was confirmed through analysis of the fluorescence output of the specimens of all the subjects. No clinical adverse events occurred during the procedure or survival phase of the experiment and no damage was observed either on neuroimaging, during or after the procedure, or on histopathological evaluation - with the exception of a single subject sustaining an injury during closure of the scalp. The multifocal areas of acute subarachnoid hemorrhage reported on histopathological analysis of certain subjects were suggestive of a terminal event and not attributable to the procedure.

Concerning brain tumors, different factors generally guide the choice of the most appropriate therapeutic approach. For example, localization is of paramount importance in brain tumor management, since deep masses are not usually well-suited for open surgery, which is potentially burdened by severe neurological morbidity. Other therapeutic strategies, such as laser interstitial therapy or radiosurgery, have shown limited results in this context (7, 26, 27). In comparison, SDT may represent an innovative modality for the treatment of intracranial masses, as it exploits the ability of certain compounds (i.e. sonosensitizers) to be activated by ultrasound irradiation, thus locally exerting their biological effect in defined targets where they selectively accumulate. To this end, the interaction between the sensitizing agent and the lesion which selectively uptakes it acts as a “self-focusing mechanism” of this non-invasive treatment modality.

It is known that, in SDT, neither the low frequency insonation nor the sensitizing agent are able to appreciably treat any lesions when used alone; however, their simultaneous application can exert a cytotoxic effect (9).

**Figure 6:**
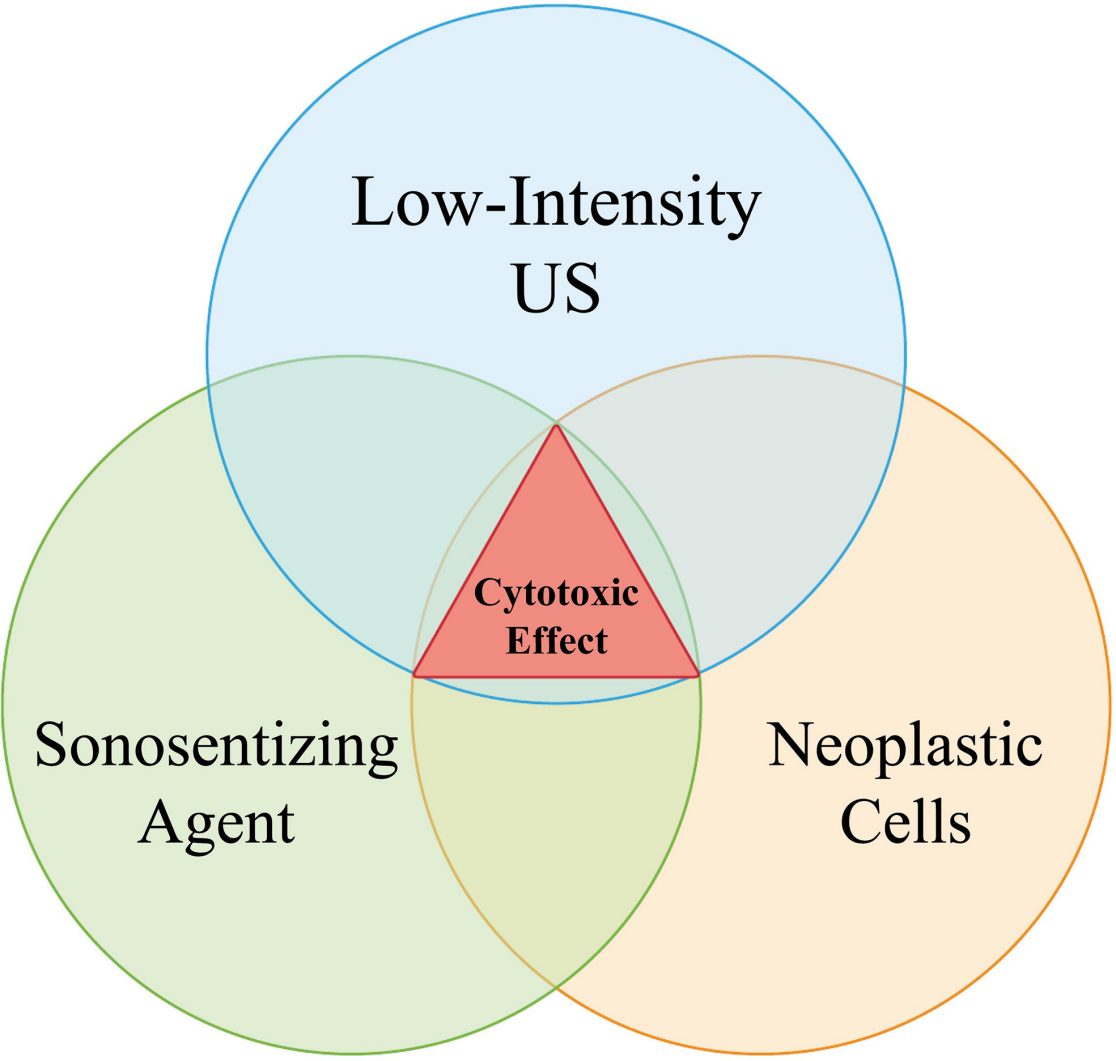
The Venn Diagram explains the relation between sonosensitizer administration (5-ALA or Na-Fl), low-frequency sonication and tumor presence. The contemporary presence of all the three aforementioned events is required for a cytotoxic process to verify. As “tumor” here it is meant any lesion which is able to properly locally concentrate the administered sonosensitizers, granting the onset of their biological effects once activated by sound beams.

This study aimed to push this paradigm one step further by demonstrating that even the simultaneous presence of both the ultrasound wave and the sensitizer is insufficient to produce significant effects, as there is the additional critical consideration of a “concentration effect” within the target lesion, be it neoplastic or otherwise. As no injuries were detected on clinical, radiological, and histopathological examinations following SDT, our results suggest that even if the presence of sonosensitizers had been confirmed in healthy brain tissues, this accumulation would be inconsequential. Indeed, in our subjects, the concentration of sensitizers was too low for determining a biological effect, since the absence of an area where these compounds can preferentially accumulate, such as a tumor. In light of this evidence, we postulate that three particular contemporary events must occur in order for SDT to render a cytotoxic effect: the administration of ultrasound and sonosensitizer and the presence of a lesion where the latter can reach a particular concentration, thus adding a further safety mechanism for SDT (**Figure 6**).

A crucial factor, which may hinder the future translation of SDT into clinical use, is the time required for the treatment; unlike sonication of small structures, as that performed, for example, for thalamic ventral intermediate nucleus (Vim) in essential tremor, the larger volumes which need to be treated in SDT for intracranial tumors require multiple sonications, and consequently, much longer treatment times. In contrast with small animals or *in vitro* models, this problem became particularly evident in the swine model employed herein.

In the present study, the time required for each procedure was drastically optimized by taking advantage of the low DC (10%) of each sonication and rastering the sonication target in 10 sub-sonication areas, thus exploiting the 90% off-time of each DC by treating 9 additional spots. As a result, the total “ON” time for the transducer was almost 100%, but each spot received only a 10% DC, as commonly used. The aforementioned approach gives rise to a new sonication scheme, enabling treatment of larger volumes in the same amount of time and could be of great importance for translating SDT from animal models to clinical practice. On the other hand, it is noteworthy that such insonation strategy does not expose subject to higher energy levels, nor does it change the way each spot is treated per se; rather it exploits the silent “breaks” of traditional schemes to speed up the whole process.

Many preclinical studies have already investigated the safety and efficacy of both 5-ALA and Na-FL. Nonetheless, published experiences have generally employed small rodent models, with only minor subsets of healthy subjects (4); furthermore, sonication devices were mostly experimental, with only one study having employed a device currently in use for human ultrasonic treatments (20). To the best of our knowledge, this was the first proof of principle for intracranial MRI-guided SDT in a large animal model using a device that is in active clinical use. The porcine model allowed a more precise target definition, in a more similar way to the technique that would be used for clinical purposes in humans. Furthermore, the employed sonosensitizers are already approved for clinical usage in brain tumor surgery.

Even if HGGs are the most common and aggressive group of primary malignant tumors of the brain, there are other types of lesions which, despite their benign histological classification, comprise a group of challenging pathologies; this is attributable to their high local recurrence, aggressive behavior or deep location, e.g. chordomas or skull base meningiomas. If PpIX, the photo- and sono-dynamically active compound of 5-ALA, is known to accumulate in HGGs due to abnormal ferrochelatase activity in glial cells, its selectivity for these other tumor types is expected to be much less pronounced. The localization of Na-Fl, on the other hand is mainly dictated by BBB alterations and has been confirmed in different tumoral contexts, including meningiomas, hemangioblastomas, metastases, ependymomas, pilocytic astrocytomas, and schwannomas (28, 29). The safety of Na-Fl SDT, therefore, may open new hints towards future studies involving the treatment of different brain tumors beyond gliomas.

This study may bear some noteworthy future directions. First clinical experiences with SDT for brain tumors will likely rely on MRgFUS devices employing a stereotaxic frame; indeed, two clinical trials in the USA and Europe (NCT04559685 and NCT04845919 respectively) employing similar parameters and sensitizers’ dosage were already approved. Future experimental designs are likely to benefit from the “rastered” insonation scheme herein proposed. This strategy will require further investigation to directly compare its efficacy with that of “classic” sonication modalities. Moving forward, SDT for brain tumors might also benefit from approaches and devices that enable more agile and repeatable approaches to the disease management (30–32).

### Limitations

The current study presents different limitations, among which the restricted sample size of the study; however, the decision to include only 9 specimens was made in order to minimize animal use by the Animal Care and Use Committee.

Furthermore, only one insonation protocol was tested in our experimental design for the same reason; however, the parameters employed in the study were derived from previous preclinical experiences (4, 19, 20, 33–36), the efficacy of which will be tested in one FDA and on CE approved clinical trial.

In this regard might sound limiting the absence of a pathological lesion in our experimental design to demonstrate concurrent effects of SDT towards it in the absence of damages to healthy tissues; it has to be kept in mind that the goal of the study was to prove that SDT does not affect brain tissue even when US beams are focused directly towards it. Of course, the present model does not indeed comprise a tumor marginal zone with compromised blood-brain barrier and potential higher concentration of the sensitizer, especially concerning Na-Fl. Furthermore, it has to be taken into account the absence of a validated porcine model of glioblastoma comparable to murine models employed in other studies. Future studies are warranted once this model will be readily available.

Tissue damage was only evaluated *via* H&E-stained histologic preparations, without further immunohistochemical or molecular assays; while these more complex methodologies are generally employed for tumor-bearing subjects (37–39), our present approach is consistent with other studies investigating safety of SDT in healthy specimens (40–42).

## Conclusions

The current study demonstrated that insonation of both 5-ALA and Na-Fl is harmless from a clinical, radiological and histopathological point of view towards healthy brain tissue in absence of a lesion that is able to concentrate the aforementioned compounds to a determined level.

The ability of ultrasound to reach deep intracerebral structures may enable the possibility to treat lesions otherwise not amenable to benefit from open surgery.

With the results of the present study, new evidence in favor of SDT safety is provided, with the aim to ease its translation from experimental studies to clinical practice; this path, however, still has some hurdles. One major concern is that ultrasound procedures generally require long treatment sessions and this confines their application to small volumes; in order to overcome this problem, we propose a novel sonication procedure that exploits the duty cycle of SDT exposure conditions to expedite treatment time.

Finally, the current study suggests a new found flexibility to adjust the SDT treatment envelope to every lesion that is able to concentrate a particular sonosensitizer, in a manner appropriate for each compound’s particular pharmacokinetic properties.

## Data Availability Statement

The original contributions presented in the study are included in the article/supplementary material. Further inquiries can be directed to the corresponding author.

## Ethics Statement

The animal study was reviewed and approved by Animal Care & Use Committee (ACUC).

## Author Contributions

Conceptualization: FP, DM, KT, and ZX. Experimental procedure: FP, DM, KT, SA, JG, AF, and ZX. Data analysis: FP, LR, AD’A, MG, and EP. Writing, review and editing: LR, AD’A, MG, NS, JG, M-BL, AF, DM, SA, JS, ZX, and FP. Supervision: FP, FD, and JS. Project administration: FP, NS, DM, KT, and ZX. Funding acquisition: FP, JS, and ZX. All authors contributed to the article and approved the submitted version.

## Funding

The present study was funded and supported by the Focused Ultrasound Foundation (Charlottesville, VA, USA).

## Conflict of Interest

The authors declare that the research was conducted in the absence of any commercial or financial relationships that could be construed as a potential conflict of interest.
